# Development and validation of a model for soil wetting geometry under Moistube Irrigation

**DOI:** 10.1038/s41598-022-06763-x

**Published:** 2022-02-17

**Authors:** T. L. Dirwai, A. Senzanje, T. Mabhaudhi

**Affiliations:** 1 Crop-, Soil-, and Climate Sciences, University of the Free State, P.O. Box 339, Bloemfontein, 9300 South Africa; 2 School of Engineering, University of KwaZulu-Natal, P. Bag X01, Pietermaritzburg, 3209 South Africa; 3 Centre for Transformative Agricultural and Food Systems, School of Agricultural, Earth and Environmental Sciences, University of KwaZulu-Natal, P. Bag X01, Pietermaritzburg, 3209 South Africa; 4 International Water Management Institute (IWMI-GH), West Africa Office, c/o CSIR, Accra, Ghana

**Keywords:** Scientific data, Environmental sciences

## Abstract

We developed an empirical soil wetting geometry model for silty clay loam and coarse sand soils under a semi-permeable porous wall line source Moistube Irrigation (MTI) lateral irrigation. The model was developed to simulate vertical and lateral soil water movement using the Buckingham pi (π) theorem. This study was premised on a hypothesis that soil hydraulic properties influence soil water movement under MTI. Two independent, but similar experiments, were conducted to calibrate and validate the model using MTI lateral placed at a depth of 0.2 m below the soil surface in a soil bin with a continuous water supply (150 kPa). Soil water content was measured every 5 min for 100 h using MPS-2 sensors. Model calibration showed that soil texture influenced water movement ($p$ < 0.05) and showed a good fit for wetted widths and depths for both soils ($nRMSE$ = 0.5–10%; $NSE \ge$ 0.50; and d-index $\ge$ 0.50. The percentage bias $\left( {PBIAS} \right)$ statistic revealed that the models’ under-estimated wetted depth after 24 h by 21.9% and 3.9% for silty clay loam and sandy soil, respectively. Sensitivity analysis revealed agreeable models’ performance values. This implies the model's applicability for estimating wetted distances for an MTI lateral placed at 0.2 m and MTI operating pressure of 150 kPa. We concluded that the models are prescriptive and should be used to estimate wetting geometries for conditions under which they were developed. Further experimentation under varying scenarios for which MTI would be used, including field conditions, is needed to further validate the model and establish robustness. MTI wetting geometry informs placement depth for optimal irrigation water usage.

## Introduction

Agriculture is the largest consumer of blue water^1^ at 70% of all global freshwater resources^2,3^. Novel irrigation technologies such as sub-surface irrigation and porous pipes promote water conservation^2^. Moistube Irrigation (MTI) is a semi-permeable porous pipe that has reported improved field water use efficiency (fWUE). MTI is a sub-surface irrigation technology whose discharge is facilitated by an applied pressure, or at zero pressure, it utilizes soil water matric potential ($\psi$) that causes a pull effect, thus facilitating discharge. MTI is a semi-permeable porous wall tubing; thus, its wetting geometry is classified as a line source emitter^4,5^. Soil water movement under various irrigation technologies has informed irrigators on the effective placement depth and lateral spacing that promote crop water use efficiency (cWUE) and fWUE.

There is limited empirical knowledge on models that facilitate the estimation of wetted perimeters under porous wall emitters. Knowledge of soil wetting geometry is critical in optimizing MTI irrigation system design (lateral placement depth and spacing) and operation (discharge rates, irrigation set times and satisfying irrigation water requirements). To maximize the advantages offered by sub-surface irrigation, knowledge of soil wetting geometries aids in irrigation network design, i.e., emitter spacing and placement depths, which subsequently improve irrigation schedules^6^, minimize run-off losses, promotes higher irrigation uniformity^7,8^, increases water productivity (WP) and fWUE^4,9^.

Soil wetting geometries can be determined either experimentally or using modelling tools. The former is expensive and time-consuming. Modelling is a time-saving exercise, and numerical models have gained wide applicability over their counterparts (analytical and empirical models) because of their robustness and use of finite element boundary approximation techniques^4,10^. Experimental and simulation models for line source semi-permeable emitters can potentially shed light on robust installation and management guidelines for MTI^11^.

Kandelous and Šimůnek^12^ conducted comparative research on analytical (WetUp), numerical (HYDRUS-2D) and empirical models’ performance on estimating wetting geometries under trickle irrigation. The models were evaluated using the mean absolute error (MAE) and the R^2^ value. The MAE ranged from 1 to 58.1 cm for WetUp, 0.87 to 10.43 cm for HYDRUS-2D, and 1.34 to 12.24 cm for the selected empirical models. The study obtained good R^2^ values that ranged from 0.71 to 0.84 for all models. Cook, Fitch^13^ assessed point source trickle irrigation wetting dimensions using HYDRUS-2D and WetUp. The findings revealed the models’ equal capacity in estimating wetted dimensions; however, HYDRUS-2D had difficulties estimating wetted dimensions for soils with low hydraulic conductivity. Kanda, Senzanje^4^ numerically and experimentally estimated soil wetting dimensions under MTI. Elmaloglou, Soulis^14^ developed a numerical model for simulation of soil water distribution under line source subsurface drip irrigation (SDI) considering hysteresis.

Despite their simplicity, empirical models can also be applied to help answer design and management questions in soil water movement and irrigation water management. Schwartzman and Zur^15^ developed an empirical model that simulated horizontal and vertical wetting geometries for line source water application under Gilat loam and Sinai sand soils. The wetting geometries depended on emitter discharge, total soil water volume and soil characteristics. Dabral, Pandey^16^ modified the Schwartzman and Zur^15^ equations and modelled the horizontal width ($W$) and vertical depth ($Z$) from a point source emitter. The models’ versatility were closely similar to those by Schwartzman and Zur^15^ and Keller and Bliesner^17^; however, a lack of the emitter placement depth variable potentially limited the accuracy of the model.

Amin and Ekhmaj^18^ developed an empirical model that estimated horizontal ($R$) and vertical downward ($Z$) distances of the wetting front from the surface drip emitter, whereas Singh, Rajput^19^ applied the dimensional analysis to determine the wetted width ($W$) and wetted depth ($D$) of sandy loam soils under subsurface drip irrigation. Kandolous, Liaghat^20^ employed Singh’s^19^ method to develop an empirical model that estimated the horizontal ($W$), vertical upward ($Z_{ + }$), and vertical downward ($Z_{ - }$) wetting distances.

The literature presents evidence on the exhaustive use of analytical and numerical models for estimating soil wetting geometries or dimensions under point source and line source surface and subsurface irrigation. However, limited literature exists on an empirical model that estimates wetting geometries under a relatively new SPM MTI porous lateral. Empirical models tend to bring about simplicity and ease of application without the need for extensive data sets and, at times, proprietary software.

MTI flow approximates a semi-permeable porous line source two-dimensional flow, and Richards’ Equation best describes the water flow process. Flow simulation models utilize the van Genuchten–Mualem constitutive relationships to estimate the soil hydraulic parameters and aid in plotting soil water retention curves that are subsequently used to determine soil water content at various water potential head ($h$). The van Genuchten–Mualem constitutive variables can be potentially incorporated in empirical model development to accommodate the soil hydraulic properties; this subsequently provides an actual representation of the soil water movement component in the flow or wetting geometry simulation.

The Buckingham $\pi$ theorem or dimensional analysis is a powerful and useful tool in developing empirical simulation models^21^. The Buckingham π theorem is premised on the concept that physical laws are independent of the units that define the variables^22^. The method simplifies processing by reducing dimensional quantities that describe physical terms into a few and manageable non-dimensional quantities called $\pi$ terms^23^. Tillotson and Nielsen^23^ applied the $\pi$ theorem to derive scale factors for soil properties, whilst studies by Singh, Rajput^19^ and Dabral, Pandey^16^ applied dimensional analysis to successfully develop soil wetting simulation models. Despite the development of empirical models for simulating soil wetting under surface and sub-surface drip irrigation using the π theorem; however, the concept has not been extended to MTI.

Whilst Kanda, Senzanje^24^ used HYDRUS 2/3D to simulate the wetting fronts of a sandy-clay-loam and loamy sand under MTI, this paper adds to the body of knowledge by developing an empirical model that simulates the horizontal and vertical wetting geometry of line source water application for a silty clay loam, and a coarse sand soil under MTI—the two soils were deliberately selected to cover the two extremes of fine-textured and coarse-textured soils, and attempt to establish the operational boundaries of the empirical model developed. Several studies^15,19,25–28^ have developed soil wetting geometry simulating models under point source or trickle source; however, there is limited knowledge on the wetting geometries of porous irrigation pipes such as MTI in particular. We hypothesized that soil hydraulic properties influenced the horizontal and vertical soil water movement under MTI. Therefore, the objective of the current study was to develop and test soil wetting empirical models’ for silty clay loam and sandy soils.

## Materials and methods

### Model description

The horizontal ($W$) and vertical ($Z$) wetting geometry of the semi-permeable porous line source water application MTI were assumed to be a function of the total volumetric soil water content per unit length of the MTI lateral ($V$), emitter discharge per unit length of MTI lateral ($q$), hydraulic conductivity of the soil ($k$), and placement depth $\left( D \right)$^19^. The relationship was modelled according to Eq. (1).

1 $$ (W,\,\,Z) = \,f(V,\,\,q,\,\,k,\,\,D) $$

Equation (1) reduces to Eq. (2)

2 $$ f(V,\,\,q,\,\,k,\,\,W,\,\,Z,\,\,D) $$

Using t Buckingham π theorem^16,19^, four πs were derived as presented in Eqs. (3)–(7). The four πs were derived because the Buckingham π theorem states that if there is a physically meaningful equation involving a certain number, n, of physical variables in a problem and these variables contain ‘m’ primary dimensions, the equation relating all variables will have (n–m) dimensionless groups. There were six variables with two primary variables, namely $W$ and $Z$, which resulted in 4 dimensionless groups.

3 $$ f(\pi_{1} ,\,\,\pi_{2} ,\,\,\pi_{3} ,\,\,\pi_{4} ) = 0 $$

where

4 $$ \pi_{1} = \frac{Z}{D} $$

5 $$ \pi_{2} = \frac{W}{D} $$

6 $$ \pi_{3} = \frac{V}{{D^{2} }} $$

7 $$ \pi_{4} = \frac{kD}{q} $$

Multiplying the $\pi_{3}$ and $\pi_{4}$ yielded the dimensionless soil water content per unit length of MTI $\left( {V^{*} } \right)$ as presented by Eq. (8).

8 $$ V^{*} = V\left( \frac{k}{qD} \right) $$

By taking the square root of the product of $\pi_{4}$ and $(\pi_{2} )^{2}$ yielded the dimensionless wetted width $(W^{*} )$ as presented by Eq. (9).

9 $$ W^{*} = W\left( \frac{k}{qD} \right)^{0.5} $$

The square root of the product of $\pi_{4}$ and $(\pi_{1} )^{2}$ yielded the dimensionless wetted depth $\left( {Z^{*} } \right)$ as presented by Eq. (10).

10 $$ Z^{*} = Z\left( \frac{k}{qD} \right)^{0.5} $$

Schwartzman and Zur^15^ and Singh, Rajput^19^ postulated that there exists a relationship amongst dimensionless parameters. For this research, the relationships are as presented in Eqs. (11) and (12);

11 $$ W^{*} = A_{1} V^{{*b_{1} }} $$

12 $$ Z^{*} = A_{2} V^{{*b_{2} }} $$

where $A_{1}$, $A_{2}$, $b_{1}$, and $b_{2}$ are constants for a 2-dimensional flow model. The constants $A_{1}$ and $b_{1}$, were determined from the graphical plot of $V^{*}$ and $W^{*}$ whereas the constants $A_{2}$ and $b_{2}$ were determined from the graphical plot of $V^{*}$ and $Z^{*}$.

Combining Eqs. (8) and (9) and Eqs. (8) and (11) yielded the wetted width ($W$) and wetted depth ($Z$) functions presented in Eqs. (13) and (14), respectively.

13 $$ W = A_{1} V^{{b_{1} }} \left( \frac{k}{qD} \right)^{{(b_{1} - 0.5)}} $$

14 $$ Z = A_{2} V^{{b_{2} }} \left( \frac{k}{qD} \right)^{{(b_{2} - 0.5)}} $$

### Experimental design and data collection

#### Soil hydraulic parameters and textural characteristics

The silty clay loam (34% clay, 58% silt, 8% sand) was obtained from the University of KwaZulu-Natal’s Ukulinga Research Farm in Pietermaritzburg, KwaZulu-Natal, South Africa (29° 39′ 44.8ʺ S 30° 24′ 18.2ʺ E, altitude: 636 m). The coarse sand soil (98% sand and 2% gravel) was obtained from Genie sand in Pinetown, KwaZulu-Natal, South Africa (29° 48′ 08.7ʺ S 31° 00′ 37.8ʺ E). Soil samples were subjected to soil textural analyses using the hydrometer method. The experiment sampled five depths for textural analysis, and the resultant textural data was fed into the SPAW model (Saxton and Willey, 2005) to determine saturated hydraulic conductivity ($k_{s}$) was derived. Other soil hydraulic parameters total porosity ($\theta_{r}$), residual soil water content ($\theta_{s}$), and shape fitting parameters ($n$, $m$, and α) (Table 1) were laboratory determined using the soil–water retention pressure method^4,29,30^. The 50 cm depth soil sample for the silty clay loam was used to fit the van Genuchten parameters because the 50 cm plot provided a smooth curvilinear shape and the resultant parameters closely aligned with Rawls, Brakensiek^31^. The sandy soil was commercially acquired hence the absence of varied sampling depths. The methods were selected based on the reliability of results and equipment availability.

**Table 1:** Soil textural and soil hydraulic parameters.

Textural class	Depth (cm)	$\theta_{r} \left( {{\text{cm}}^{3} {\text{cm}}^{ - 3} } \right)$	$\theta_{s} \,\, \left( {{\text{cm}}^{3} {\text{cm}}^{ - 3} } \right)$	$n$	$k_{s} \,\,\,\left( {{\text{cm}} {\text{day}}^{ - 1} } \right)$	$\alpha$	$m$	BD (g cm^−3^)
Silty clay loam	10	0.11	0.52	1.65	62.67	0.01	0.39	0.52
Silty clay loam	20	0.267	0.58	6	62.67	0.01	0.83	1.10
Silty clay loam	30	0.4	0.49	7	62.67	0.01	0.85	1.36
Silty clay loam	40	0.04	0.51	6.5	62.67	0.011	0.85	1.29
Silty clay loam	50	0.04	0.59	5	62.67	0.01	0.08	1.36
Sand	NIL	0.020	0.54	2.68	513.21	0.03	0.63	1.22

*BD* bulk density.

#### Measurement of soil wetted front

The soil was air-dried, crushed and sieved through a 2 mm sieve. Thereafter, the soil was loaded into a soil bin measuring 1 m (H) × 1 m (W) × 0.5 m (B). The soil bin had transparent Plexiglass walls, and soil loading was done gently to avoid compaction and possible crushing of the MTI tubing. To prevent MTI collapse under the soil surcharge, MTI was supplied with water before loading the soil till it was turgid. To prevent MTI smearing and potential nano-pore blocking, the water was supplied upon reaching the MTI burying level. The MTI lateral was placed at a depth of 0.2 m below the soil surface, and upon soil loading, MPS-2 sensors were simultaneously installed at prescribed depths (Table 2). The initial soil–water content for the silty clay loam was 1.02 × 10^–6^ m^3^ and 6.64 × 10^–7^ m^3^ for the sandy soil. Both soils were packed at a bulk density of 1.4 g cm^−3^.

**Table 2:** MPS-2 sensors placement depths and lateral spacing for the respective soils.

Soil texture	MPS-2 placement depth (m)	MPS-2 lateral spacing (m)
Silty clay loam	0.1, 0.2, 0.25,and 0.3	0.05, 0.15, 0.20, and 0.25
Sand	0.1, 0.15, 0.2, 0.3, 0.4, 0.5, 0.6, 0.7, and 0.8	0.1, 0.2, and 0.3

The MPS-2 sensors measured water potential (− 10 to − 500 kPa) and temperature, and they were calibrated by soaking them in de-ionised water for a period of 72 h before installation. The de-ionised water was used to substitute for the conventional mercury porosimeter experiment for calibration. The calibration curve was obtained from the MPS-2 and MPS-6 operators manual (Decagon Devices Inc, 2017). Water to the MTI lateral was supplied at a pressure head of 150 kPa, which discharged 2.39 l h^−1^ m^−1^ (Table 3).

**Table 3:** Models’ inputs.

Parameter	Value	
	Silty clay loam soil	Sandy soil
$k$	62.67 cm day^−1^	513.21 cm day^−1^
$q$	2.39 l h^−1^ m^−1^	2.39 l h^−1^ m^−1^
$D$	0.2 m	0.2 m
$V$	Volumetric soil water content	
$W$ and $Z$	Measured physically	

**Figure 1:**
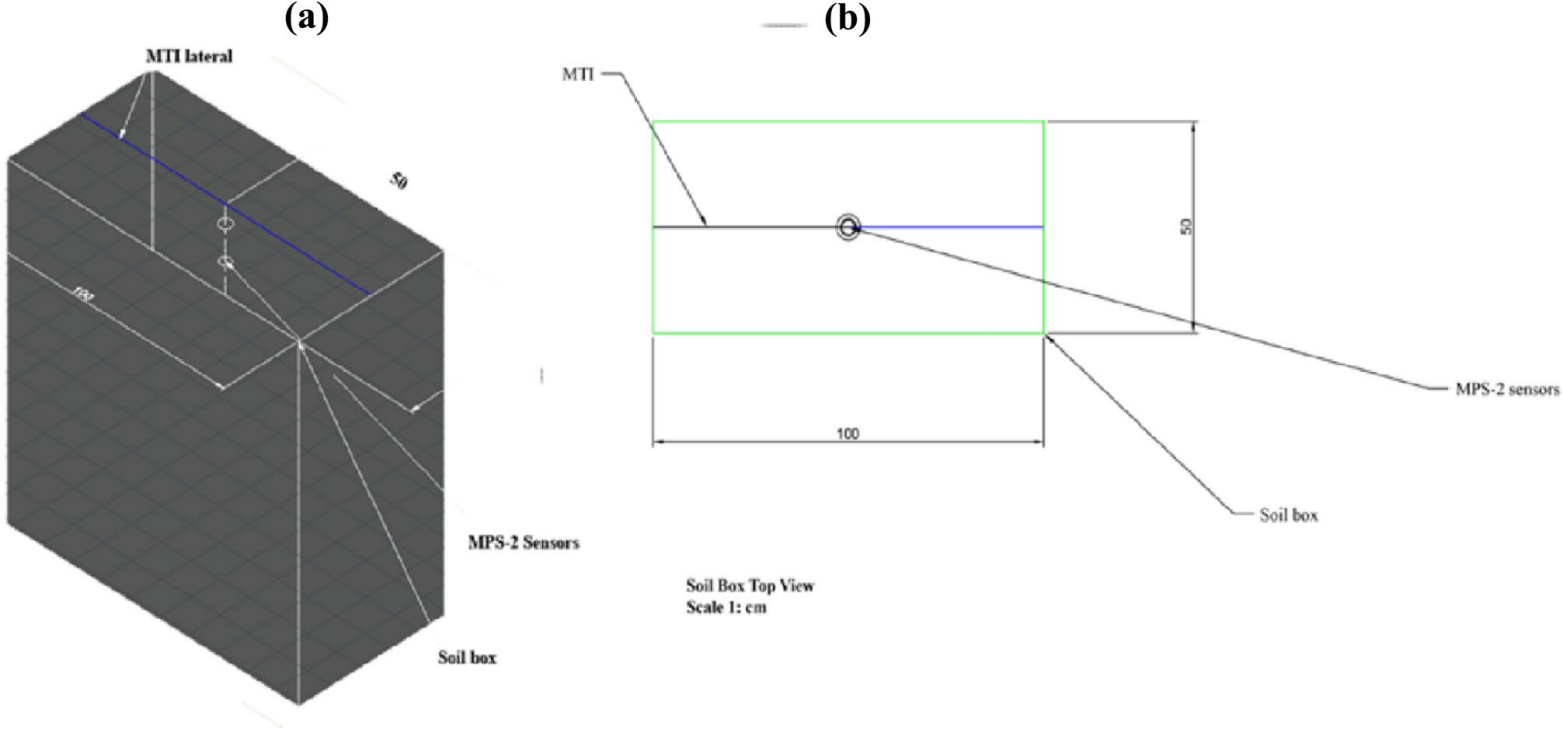
Soil bin experimental set-up (**a**) 3-D view and (**b**) orthographic top view. Dimensions in cm.

The experiment was carried out in two phases. The first dataset of measured variables was used for model calibration, whilst the measured variables from the second phase dataset were used for model validation. The measured variables are summarised in Table 4. Both the first and second phases were carried under identical conditions. Soil water-retention curves derived from the soil–water retention experiment were used to determine the volumetric soil water content. The experimental equipment set-up is shown in Fig. 1.

**Table 4:** The experiments used for models’ calibration and validation.

Phase	Description	Use
1	Determining the constants $A_{1}$, $A_{2}$, $n_{1}$ and $n_{2}$	Calibration
2	Testing the models’ predictive capacity	Validation

### Models’ calibration

The models’ calibration was done in the first phase of the experiment. The calibration process was done to determine the models’ constants $A_{1}$, $A_{2}$, $n_{1}$ and $n_{2}$. To improve the models’ precision and accuracy, iterations were carried out on constants $A_{1}$ and $A_{2}$.

### Models’ validation

The parameter variability–sensitivity analysis (PV–SA) method was employed to validate the models (Sargent, 2013). A sensitivity analysis was carried out to assess the effects of $k$, $q$, and $D$ on both $W$ and $Z$. The sensitivity analysis was done by holding all the other variables constant and assessing the functional relationship a particular “active variable” had with $W$ and $Z$. Table 4 summarise the two independent experimental phases and their respective purposes.

### Models’ evaluation

The study applied the following criteria; normalised root mean square error ($nRMSE$), index of agreement (d), Nash–Sutcliffe efficiency (NSE), and percentage bias ($PBIAS$) for the assessment of the empirical models’. The selected criteria are defined by Eqs. (15)–(18)^4,32^. Moriasi, Arnold^33^ recommended the NSE and $PBIAS$ for model evaluation because of their robust performance rating of simulating models.

15 $$ nRMSE = \frac{{\sqrt {(\frac{1}{x}} \mathop \sum \nolimits_{i = 1}^{x} \left( {O_{i} - P_{i} } \right)^{2} )}}{{O_{mean} }} $$

16 $$ NSE = 1 - \left[ {\frac{{\mathop \sum \nolimits_{i = 1}^{x} \left( {O_{i} - P_{i} } \right)^{2} }}{{\mathop \sum \nolimits_{i = 1}^{x} \left( {O_{i} - O_{mean} } \right)^{2} }}} \right] $$

17 $$ PBIAS = \frac{{\mathop \sum \nolimits_{i = 1}^{x} \left( {O_{i} - P_{i} } \right)*100}}{{\mathop \sum \nolimits_{i = 1}^{x} O_{i} }} $$

where $O_{i}$ and $P_{i}$ = observed and predicted value(s), respectively, $\overline{O}_{i}$ = mean observed data, and $x$ = number of observations. $nRMSE$ defined the developed model’s accuracy whilst $PBIAS$ defined the bias provided by the developed model. The error index *nRMSE* showed the model's performance but did not indicate the degree of over or under-estimation hence the use of the *NSE* and $PBIAS$ statistical tools in the analysis. The NSE statistic measured the residual variance vs the measured data variance, ranging from $- \infty$ to 1. NSE values between 0.0 and 1.0 are considered acceptable.

$PBIAS$ measured the tendency of the simulated data to either under- or over-estimate the observed values. Low magnitudes presented optimal model simulation whilst positive values represented model under-estimation, and negative values represented model over-estimation^33^. The study further employed the prediction efficiency ($P_{e}$). The $P_{e}$ was determined by the $R^{2}$ values obtained by regressing the observed and simulated values. A summarised performance rating for the recommended statistics is shown in Table 5.

**Table 5:** General performance rating for model evaluation statistics.

Performance rating	NSE	${\text{PBIAS}}$ (%)	Days
Very good	0.75 < NSE < 1.00	${\text{PBIAS}} < \pm 15$	0.8 < day < 1.0
Good	0.65 < NSE < 0.75	$\pm 15 < {\text{PBIAS}} < \pm 30$	0.6 < day < 0.8
Satisfactory	0.50 < NSE < 0.65	$\pm 30 < {\text{PBIAS}} < \pm 55$	0.3 < day < 0.6
Unsatisfactory	NSE $\le$ 0.50	${\text{PBIAS}} \ge \pm 15$	Day < 0.2

### Scenarios

The developed models were used for situational analysis. The MTI placement depth was maintained at 0.2 m. The operational discharge was varied between 0.27 and 3.19 l h^−1^ m^−1^, which translated to an operating pressure range of 20–160 kPa. The selected discharges used for scenario analysis were selected on the basis that MTI is a low pressure continuous discharge technology (Kanda et al. 2020). The study selected the minimal and maximum possible discharge rates to test the developed models.

## Results and discussion

### Model calibration

The calibration steps for the four wetting geometry models’ are outlined below.

#### Silty clay loam soil

The study followed the outlined steps below to determine the values for the constants $A_{1}$, $A_{2}$, $b_{1}$, and $b_{2}$^19^. Recorded volumetric soil water content and observed wetted distance values were used to calibrate the developed soil wetting geometry models. The wetted $W$ and $Z$ were physically measured using grids demarcated onto the transparent plexiglass whilst the volumetric soil water content was measured using the MPS-2 sensors.

Step 1: The dimensionless variables $V^{*}$, $W^{*}$, and $Z^{*}$ were estimated using Eqs. (8)–(10) utilising observed values of the requisite variables from the soil bin experiments.

**Figure 2:**
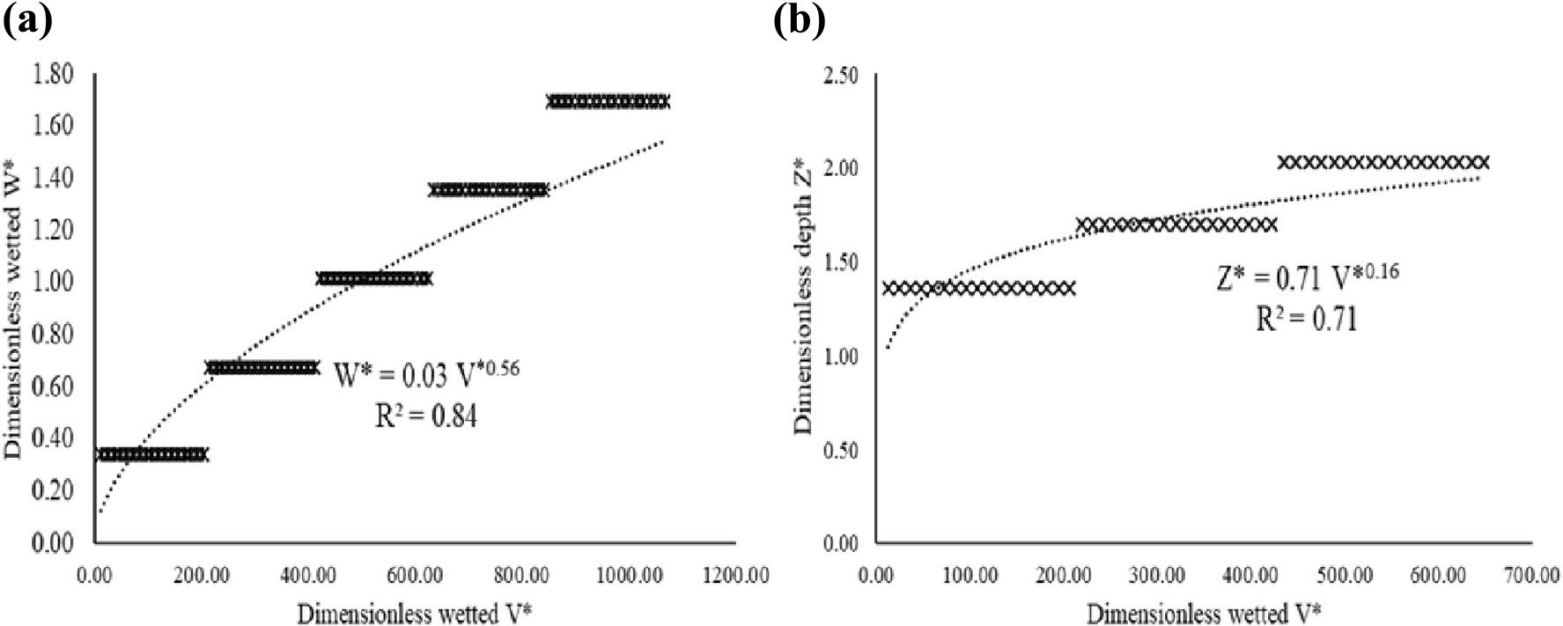
Relationship between (**a**) dimensionless wetted volume ($V^{*}$) and dimensionless wetted width ($W^{*}$) and (**b**) dimensionless wetted volume (V*) and dimensionless wetted depth ($Z^{*}$) for silty–clay-loam (the bars represented a cluster of data points).

Step 2: $W^{*}$ was plotted against $V^{*}$, similarly $Z^{*}$ was plotted against $V^{*}$ and the resultant power functions yielded Eqs. (18) and (19), with $R^{2}$ = 0.84 and $R^{2}$ = 0.71, respectively (see Fig. 2).

18 $$ W^{*} = 0.03V^{*0.56} $$

19 $$ Z^{*} = 0.71V^{*0.16} $$

From Eqs. (18) and (19), values for $A_{1}$ = 0.03, $A_{2}$ = 0.71, $b_{1}$ = 0.56, and $b_{2}$ = 0.16 were derived, and these were inputted into Eqs. (13) and (14) to yield Eqs. (20) and (21).

20 $$ W = 0.03V^{0.56} \left( \frac{k}{qD} \right)^{0.06} $$

21 $$ Z = 0.71V^{0.16} \left( \frac{k}{qD} \right)^{ - 0.34} $$

In order to improve the models’ accuracy and precision, the calibration step performed iterations on the constants $A_{1}$ and $A_{2}$ and the resultant equations are shown in Eqs. (22) and (23).

22 $$ W = 0.09V^{0.56} \left( \frac{k}{qD} \right)^{0.06} $$

23 $$ Z = 0.59V^{0.16} \left( \frac{k}{qD} \right)^{ - 0.34} $$

For the silty clay loam soil, $b_{1} > b_{2}$. This signified a high-water content in the lateral direction as compared to the vertical direction. This is typical in fine-textured soils (Bouma, 1984). Conversely, the calibration results yielded a scenario where $A_{1} < A_{2}$, which subsequently resulted in a $W < Z$, an observation atypical of fine-textured soils. This was because the application times during the experiment promoted the border effect within the confined soil bin.

#### Sandy soil

**Figure 3:**
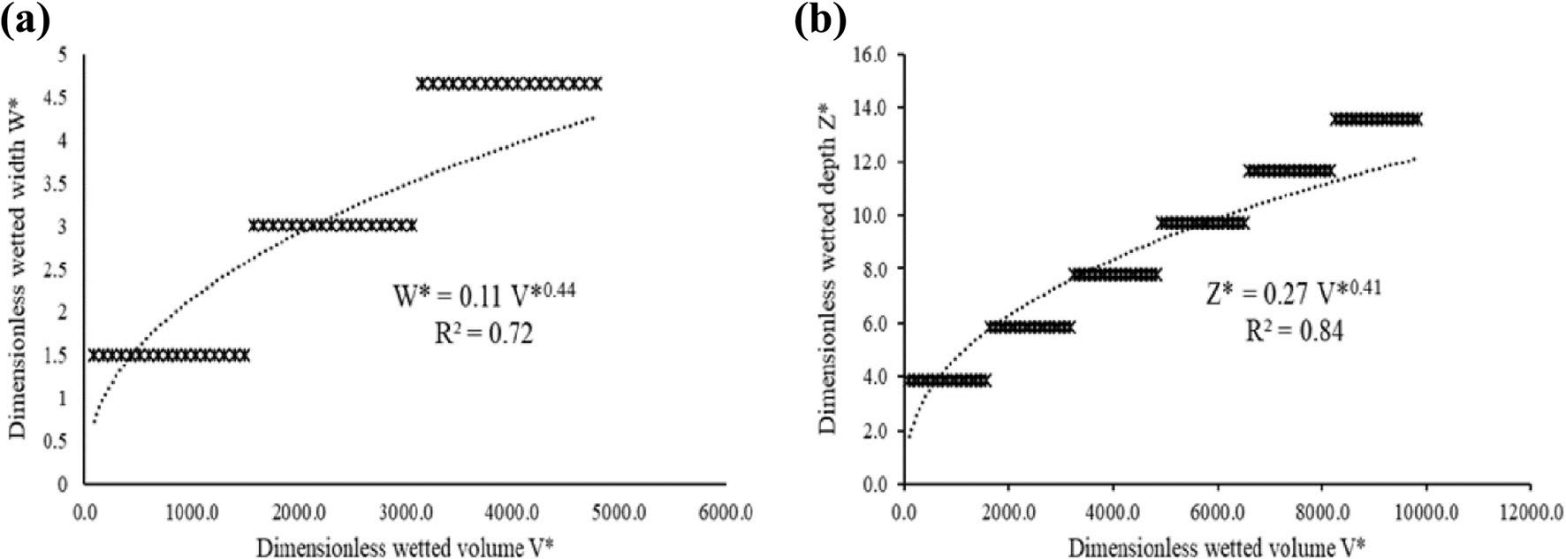
Relationship between (**a**) dimensionless wetted volume ($V^{*}$) and dimensionless wetted width ($W^{*}$) and (**b**) dimensionless wetted volume (V*) and dimensionless wetted depth ($Z^{*}$) for sandy soil.

The simulation steps for the sandy soil followed similar steps as those described for the silty clay loam soil. The relationships between the dimensionless volumetric soil water content per unit length of MTI ($$V^{*}$$) and the dimensionless wetted width ($$W^{*}$$) and wetted depth ($$Z^{*} )$$ are depicted in Fig. 3. The resultant wetted width ($$W$$) and wetted depth ($$Z$$) for the soil are shown in Eqs. (21) and (22).

$W^{*}$ was plotted against $V^{*}$, similarly $Z^{*}$ was plotted against $V^{*}$ and the resulting power relationships yielded Eqs. (24) and (25) with $R^{2}$ = 0.72 and $R^{2}$ = 0.84, respectively (see Fig. 3).

24 $$ W^{*} = 0.11V^{*0.44} $$

25 $$ Z^{*} = 0.27V^{*0.41} $$

Similarly, from Eqs. (20) and (21), the values for $A_{1}$ = 0.11, $A_{2}$ = 0.27, $b_{1}$ = 0.44, and $b_{2}$ = 0.41, respectively, we obtained, and these were inputted into Eqs. (13) and (14) to yield Eqs. (26) and (27).

26 $$ W = 0.11V^{0.44} \left( \frac{k}{qD} \right)^{ - 0.06} $$

27 $$ Z = 0.27V^{0.41} \left( \frac{k}{qD} \right)^{ - 0.09} $$

In order to improve the models’ accuracy and precision, the calibration step performed iterations on the constants $A_{1}$ and $A_{2}$ and the resultant equations are shown in Eqs. (28) and (29).

28 $$ W = 0.07V^{0.44} \left( \frac{k}{qD} \right)^{ - 0.06} $$

29 $$ Z = 0.92V^{0.41} \left( \frac{k}{qD} \right)^{ - 0.09} $$

The power indices for the sandy soil, $b_{1}$ and $b_{2}$ were approximately equal. However, the constant $A_{1} < A_{2}$, which resulted in a $Z > W$, a phenomenon attributed to soil hydraulic characteristics. Gravity forces dominated the soil water movement mechanism in the coarse-textured soil.

It is worth noting that the data in Figs. 2 and 3 were modelled against linear, logarithmic and power functions, and the power function produced satisfactory results based on the respective $R^{2}$ values. Thabet and Zayani^34^ and Dabral, Pandey^16^, in their respective soil wetting geometry studies, opted for power functions to relate $W$, $Z$ and $V$.The findings were also consistent with other MTI wetting front and infiltration studies^35–37^.

### Sensitivity analysis

To evaluate the sensitivity of the soil wetted width ($W$) and wetted depth ($Z$) to $k$ the parameters $V$, $q$ and $D$ were kept constant. This yielded Eqs. (30) and (31).

30 $$ {\text{Silty clay loam soil}}\left\{ {\begin{array}{*{20}c} {W\sim k^{0.06} } \\ {Z\sim k^{ - 0.34} } \\ \end{array} } \right. $$

31 $$ {\text{Sandy soil}}\left\{ {\begin{array}{*{20}c} {W\sim k^{ - 0.06} } \\ {Z\sim k^{ - 0.09} } \\ \end{array} } \right. $$

Considering Eq. (31), a decrease in $k$ by order of magnitude, i.e., migrating to fine-textured soil, yielded a 24% decrease in $W$ and a 119% increase in $Z$. Likewise, in Eq. (32), a decrease in $k$ by order of magnitude, i.e., migrating to fine-textured soils, resulted in approximately 15% increase in $W$ and an approximately 23% increase in $Z$.

To assess the sensitivity of the soil wetting geometry with respect to discharge ($q$), the parameters $V$, $D$, and $k$ are held constant, and the resultant relationships are outlined by Eqs. (32) and (33).

32 $$ {\text{Silty clay loam soil}}\left\{ {\begin{array}{*{20}c} {W\sim q^{ - 0.06} } \\ {Z\sim q^{0.34} } \\ \end{array} } \right. $$

33 $$ {\text{Sandy soil}}\left\{ {\begin{array}{*{20}c} {W\sim q^{0.06} } \\ {Z\sim q^{0.09} } \\ \end{array} } \right. $$

Doubling $q$ for the silty clay loam soil resulted in a 5% decrease in $W$ and a 27% increase in $Z$. Similarly, for the sandy soil when $q$ was doubled, there was a 4% increase in $W$ and a 6% increase in $Z$. Table 6 presents a summarized sensitivity evaluation containing hypotheticals and the resultant wetted horizontal and vertical wetted distances. MTI exhibited an increase in both $W$ and $Z$ in sandy soil whilst it exhibited an increase in $Z$ and a decrease in $W$ for silty clay loam. Regarding the silty clay loam soil, the findings contradict Schwartzman and Zur^15^, who posited that an increase in $q$ results in an increase in $W$ and a decrease in $Z$ of a Gilat loam soil under sub-surface drip irrigation. The difference in behaviour is attributed to the porous nature of MTI, wherein discharge is not of a point source nature.

**Table 6:** Sensitivity analysis evaluation of $$W$$ and $$Z $$ to model parameters.

Equations	Adjusting parameters						Wetted distances			
	$k_{0}$ (cm day^−1^)	$\Delta k$ (cm day^−1^)	$q_{0}$ (cm^3^ s^−1^)	$\Delta q$ (cm^3^ s^−1^)	$D_{0}$ (cm)	$\Delta D$ (cm)	$W_{o}$ (cm)	$\Delta W$ (cm)	$Z_{o}$ (cm)	$\Delta Z$ (cm)
Equation (31)	100	10	–	–	–	–	1.15	NC	1	1.19
Equation (32)	100	10	–	–	–	–	1	1.15	1	1.23
Equation (33)	–	–	1	2	–	–	1	NC	1	1.27
Equation (34)	–	–	1	2	–	–	1	1.04	1	1.06
Equation (35)	–	–	–	–	20	30	1.10	1.07	0.59	0.90
Equation (36)	–	–	–	–	20	30	1	1.04	1	1.06

$k_{o}$ initial hydraulic conductivity, $\Delta k$ order of magnitude incremental hydraulic conductivity, $q_{o}$ starting discharge, $\Delta q$ incremental discharge, $D_{o}$ initial MTI placement depth, $\Delta D$ change in MTI placement depth, $W_{o}$ initial wetted width $\Delta W$ change in soil wetted width, $Z_{o}$ initial wetted depth, $\Delta Z$ change in soil wetted width, *NC* no significant change.

The sensitivities of $W$ and $Z$ to placement depth $D$ for the respective soils was characterised by Eqs. (34) and (35).

34 $$ {\text{Silty clay loam}}\left\{ {\begin{array}{*{20}c} {W\sim D^{ - 0.06} } \\ {Z\sim D^{0.34} } \\ \end{array} } \right. $$

35 $$ {\text{Sandy soil}}\left\{ {\begin{array}{*{20}c} {W\sim D^{0.06} } \\ {Z\sim D^{0.09} } \\ \end{array} } \right. $$

An increase in $$D$$ by a unit magnitude (from 0.2 to 0.3 m) for the silty clay loam soil resulted in an approximately 3% decrease in $$W$$ and an approximately 53% increase in $$Z$$. For the sandy soil, a unit increase in $$D$$ resulted in a 4% increase in $$W$$ and a 6% increase in $$Z$$. According to Bresler^38^ $$W $$ increases for low $$k$$ values (fine textured soils) and $$Z$$ increases by a high magnitude for soils with a high $$k$$ value (coarse textured soils).

To gauge the sensitivity of $W$ and $Z$ to *V*, the parameters $D$, $q$ and $k$ were assumed constant. This yielded Eqs. (36) and (37).

36 $$ {\text{Silty clay loam soil}}\left\{ {\begin{array}{*{20}c} {V\sim W^{1.79} } \\ {Z\sim V^{0.16} } \\ \end{array} } \right. $$

37 $$ {\text{Sandy soil}}\left\{ {\begin{array}{*{20}c} {V\sim W^{2.27} } \\ {Z\sim V^{0.41} } \\ \end{array} } \right. $$

Doubling $V$ under silty clay loam soil resulted in a 47% increase in $W$ and an approximately 12% increase in $Z$. For the sandy soil, a doubling $V$ resulted in a 36% increase in $W$ and a 33% increase in $Z$.

### Models’ validation

**Figure 4:**
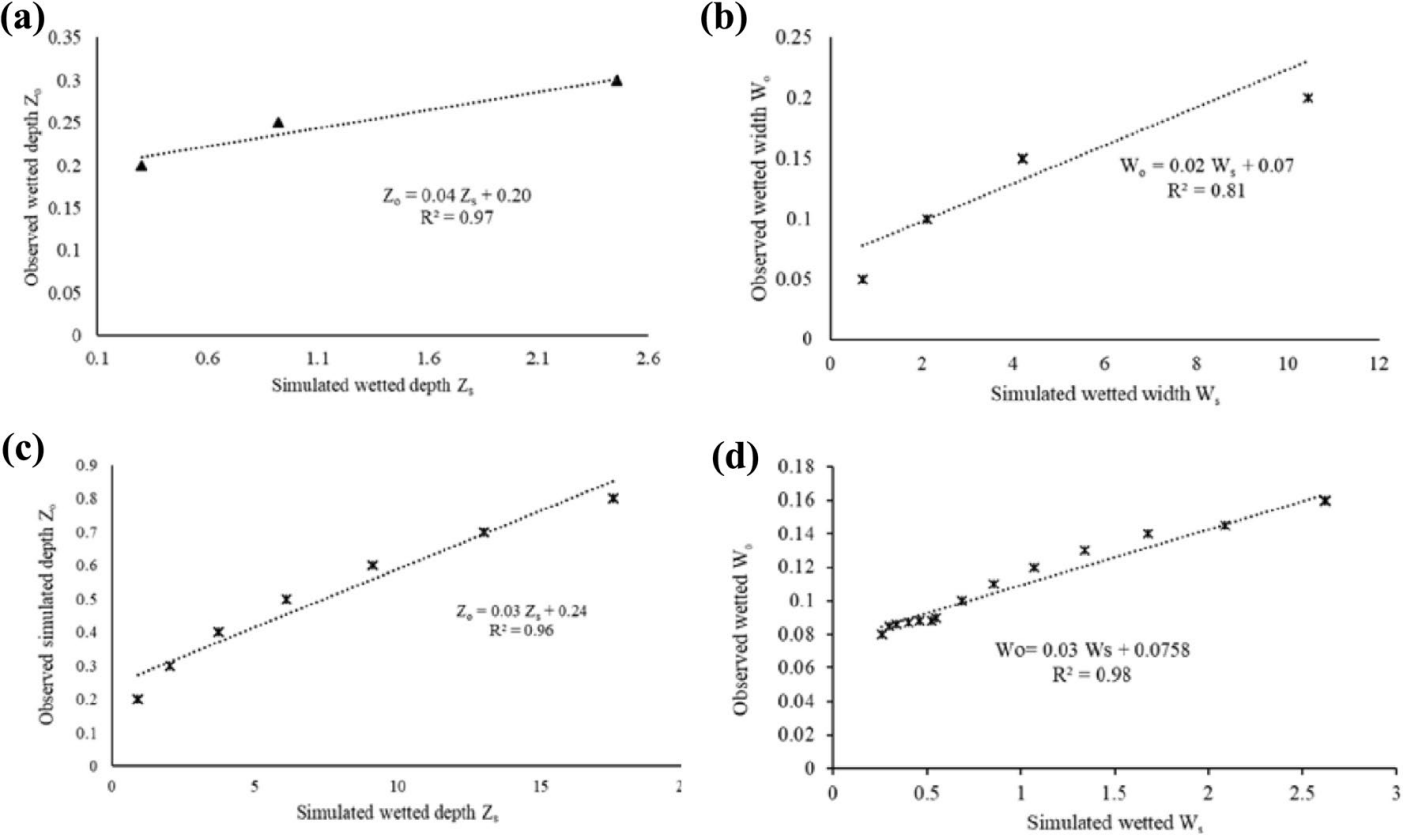
Comparison of observed $W_{o}$ and $Z_{o}$ vs simulated $W_{s}$ and $Z_{s}$ from estimated $V$ for silty clay loam (**a**,**b**) and sandy soil (**c**,**d**).

In order to obtain simulated wetted distances ($W$ and $Z$), an estimation of a range of values for $V$ was made using the sensitivity analysis relationships in Eqs. (36) and (37), and the resultant simulated $Z$ and $W$ for the semi-permeable porous wall line source 2-D flow model were computed. A correlation test based on the $R^{2}$ was carried out on a plot of simulated $W$ and $Z$ against observed $W$ and $Z$ (Fig. 4). The correlation coefficients $R^{2} > 0.75$ showed a good agreement between the observed and simulated.

The silty clay loam soil exhibited a wetting pattern on the soil surface, so for that reason, the experimental data from the MPS-2 sensor buried at 0.1 m was excluded. The soil surface wetting phenomenon was observed in both phases of the experiment. A similar observation was made by Kanda, Senzanje^4^ for an MTI tubing buried at a depth of 0.2 m. Fan, Huang^39^ also made a similar observation and posited that shallow buried depth facilitates upward water movement, a phenomenon observed in fine-textured soils.

**Figure 5:**
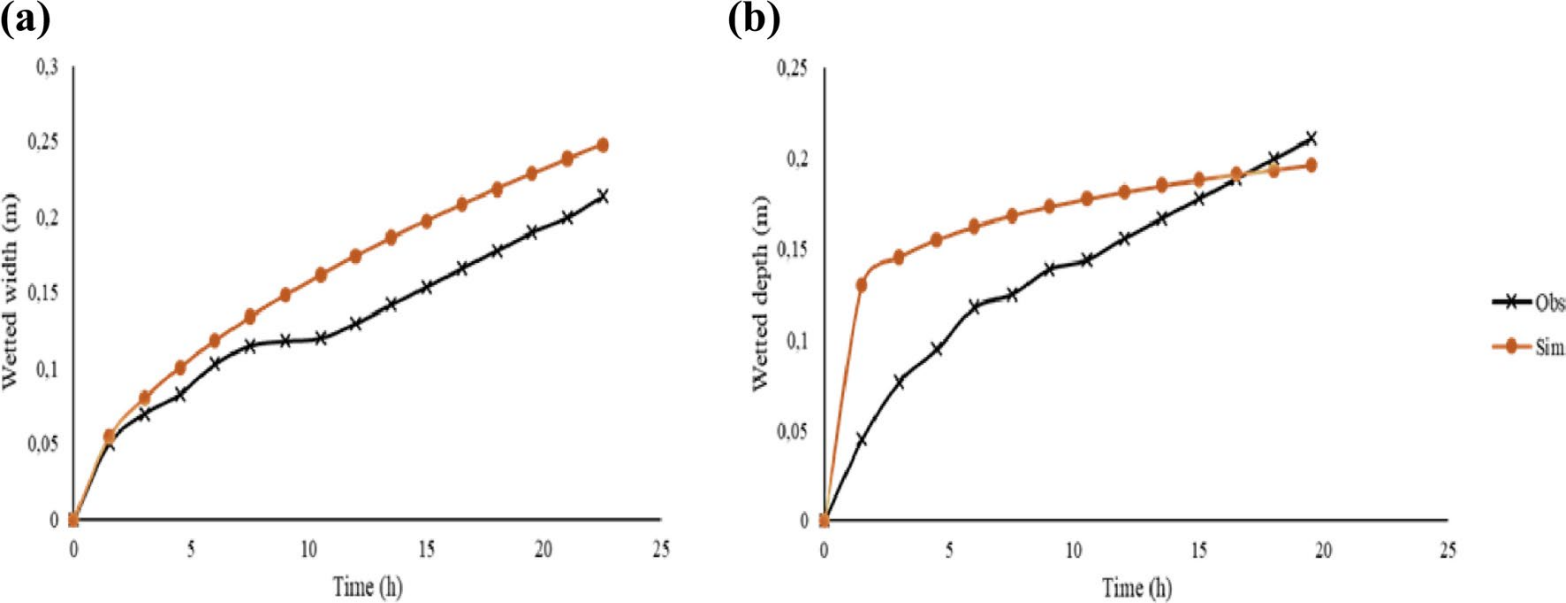
Observed vs simulated wetted distances for silty clay loam soil.

**Figure 6:**
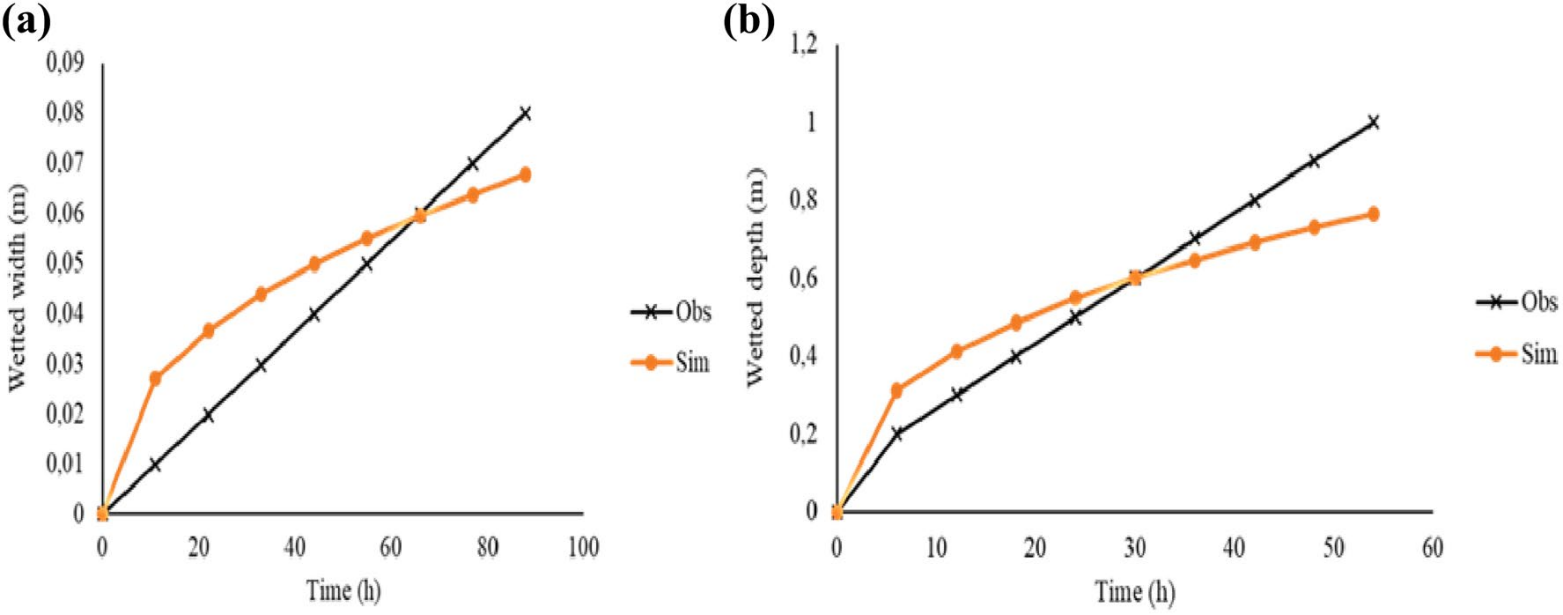
Observed vs simulated wetted distances for sandy soil.

The models' evaluation revealed a satisfactory performance. For instance, the silty clay loam models’ had a $$nRMSE$$ of 0.84% and 8.80% for $$W$$ and $$Z$$, respectively, a $$NSE$$ > 0.5, a $$PBIAS$$ < $$\pm$$ 25% and an index of agreement ($$d$$) of 1 and 0.98 for $$W$$ and $$Z,$$ respectively (Figs. 5 and 6). The sandy soil exhibited a satisfactory performance as evidenced by a $$nRMSE$$ of 0.3% and 2.5% for $$W$$ and $$Z$$ respectively, a $$NSE$$ > 0.75, a PBIAS < $$\pm$$ 15% and an index of agreement (d) of 0.6 and 0.3 for $$W$$ and $$Z,$$ respectively. The model underestimated the wetted depth ($$Z$$) for the sandy soil while overestimating the wetted width ($$W$$). A one-way ANOVA revealed a statistically significant difference ($$p$$ < 0.05) in both the observed and simulated wetted $$W $$ and $$Z$$ under sandy soil. For the silty clay loam soil, there was no statistically significant difference ($$p$$ > 0.05) between observed $$W$$ and simulated $$W$$; similarly there was no statistically significant difference ($$p$$ > 0.05) between observed $$Z$$ and simulated $$Z$$.

### Scenario analysis

#### Silty clay loam

**Figure 7:**
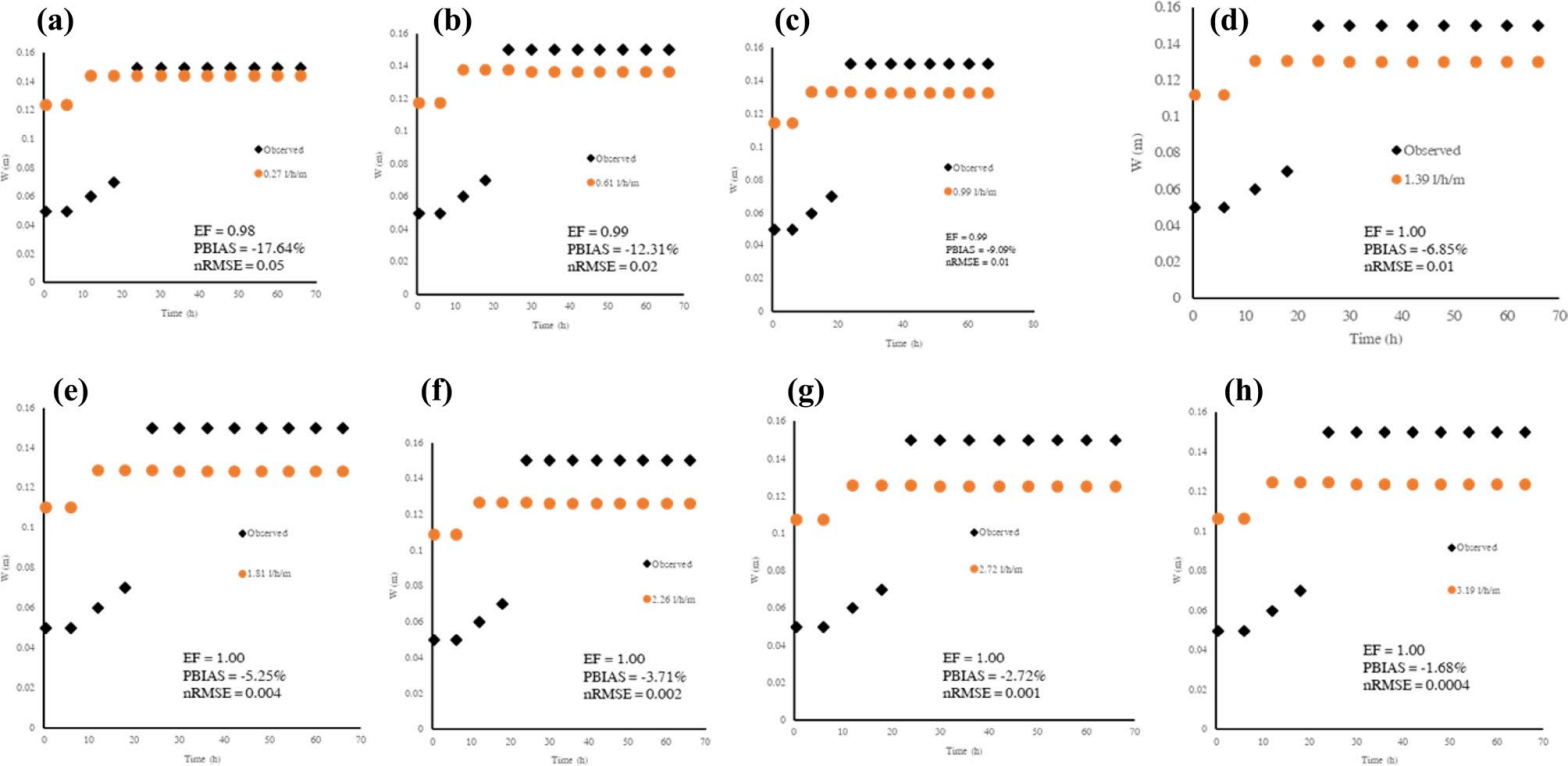
Observed vs Simulated wetted widths for silty clay loam at operating head of: (**a**) 20 kPa, (**b**) 40 kPa, (**c**) 60 kPa, (**d**) 80 kPa, (**e**) 100 kPa, (**f**) 120 kPa, (**g**) 140 kPa, and (**h**) 160 kPa.

**Figure 8:**
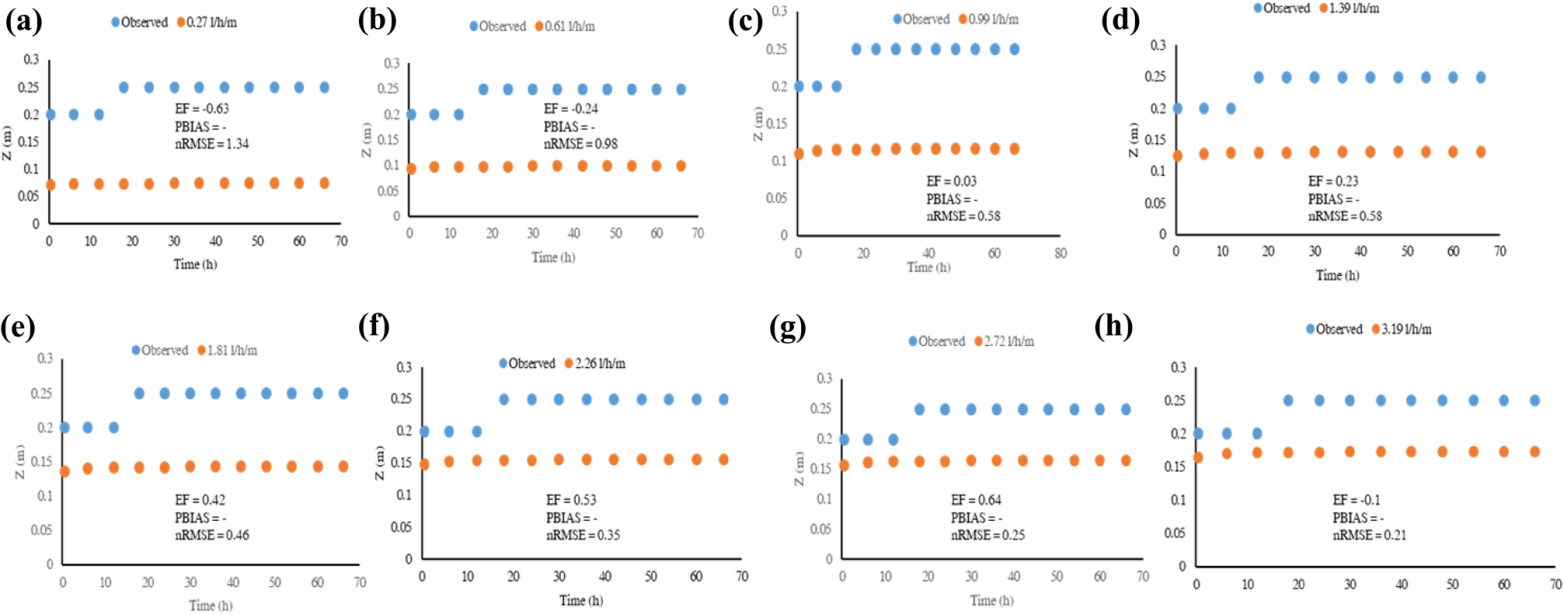
Observed vs Simulated wetted depths for silty clay loam at operating head of: (**a**) 20 kPa, (**b**) 40 kPa, (**c**) 60 kPa, (**d**) 80 kPa, (**e**) 100 kPa, (**f**) 120 kPa, (**g**) 140 kPa, and (**h**) 160 kPa.

Soil texture influenced water movement ($p$ < 0.05). Under the silty clay loam soil, the models over-estimated the wetted width ($PBIAS$ $\le 18\%$), which signified a satisfactory model performance. Other model evaluation metrics (nRMSE $\le 0.05$, EF $< 0.5$) revealed a good model performance for the wetted (Fig. 7). Interestingly, the model produced a very good agreement between the observed and simulated results at operating pressures of 80–160 kPa. The anticipated result was a good agreement at the manufacturer’s design operating pressures of 20–60 kPa. This observation can be potentially attributed to the nature of the Ukulinga soil used for the experiment. The wetted depth (Z) model performed poorly at lower discharges and operating pressures (20–100 kPa). It exhibited a relatively satisfactory performance at operating pressures of 120 kPa and 140 kPa, which translated to a discharge of 2.26 l h^−1^ m^−1^ and 2.71 l h^−1^ m^−1^ (Fig. 8). Overall, the silty clay loam soil exhibited a pronounced lateral geometry compared to the vertical geometry. The finding concurs with Fan, Huang^39^, who postulated that wetted depth is low in high clay content soils. In addition, a wetting front experiment by Cote, Bristow^11^ attributed the fine-textured soil wetting pattern to the dominance of capillarity and matric tension. Saefuddin, Saito^40^ observed pronounced radial soil water movement in a silt profile under a multiple outlet ring-shaped emitter. Fan, Huang^39^, in their MTI wetting front experiment, observed a slow lateral water movement after initial wetting of the silt-loam soil after 48 h, much similar to this study timeline.

#### Sandy soil

Granular soils exhibit a high vertical water movement due to granular pores and dominant gravitational forces; thus, the sandy soil had a greater wetted depth than the silty clay loam soil. The findings concur with a study by Cote, Bristow^11^, who performed a wetting front experiment with a sandy soil under a trickle source emitter. Ghumman, Iqbal^41^ attributed the high wetted depths in sandy soils to porosity. Although this study used a line source porous pipe, the findings relate due to the soil hydraulic characteristics factor. Another attribute to high $Z$ in sandy soils is the low macroscopic capillary length in sand which favours gravity as compared to lateral or upward movement. Siyal and Skaggs^42^ observed a similar phenomenon in modelling a sandy soil using HYDRUS 2D. The model poorly simulated the wetting distributions under an operating pressure range of 20–160 kPa (Table 7).

**Table 7:** Model performance statistics for the sandy soil models.

	Wetted width			Wetted depth		
	NSE	PBIAS (%)	nRMSE (m)	NSE	PBIAS (%)	nRMSE (m)
**Discharge (l h**^**−1**^** m**^**−1**^**)**						
0.27	0.22	51.17	0.30	–	− 7.60	0.03
0.61	0.22	49.73	0.29	–	− 15.79	0.13
0.99	0.33	48.67	0.27	–	− 20.95	0.25
1.39	0.37	47.90	0.27	–	− 24.78	0.33
1.81	0.40	47.12	0.26	–	− 27.63	0.41
2.26	0.42	46.61	0.25	–	− 30.48	0.50
2.71	0.44	46.07	0.25	–	− 32.37	0.56
3.19	–	–	1.16	–	− 34.41	0.64

### Irrigation implication

The developed soil wetting geometry models can be adopted to estimate soil wetting geometries for particular soils in question, thus ensuring optimal placement depth and spacing of MTI laterals. Knowledge of wetting geometries can potentially aid irrigators in adopting optimal lateral placement depth and spacing. The wetting depth models can inform irrigators on irrigation application times, thus availing optimal volume to the root zone and minimizing deep percolation and soil water loss due to evaporation. For fine-textured soil, shallow buried depth avails water to the soil surface that will be lost due to soil evaporation. The lateral water front for fine-textured soils expanded faster than that of coarse-textured soils; hence lateral placement under fine texture soils should be strategically placed to promote an optimal overlap between row crops. For coarse texture, close lateral placement will be required to create an optimal wetting front overlap.

### Limitations

Application of Eqs. (20)–(21) and (24)–(25) may require testing in different geographical locations and assess their universal suitability. The study was conducted for 20 h under a silty clay loam and 96 h for the sandy soil. The soil bin’s lateral dimension (width) limited the testing times for silty clay loam. The implication was the influence of border effects on soil water movement if the experimental times went beyond 20 h. Likewise, for the macroscopic sandy soil, the experimental times were limited to 96 h because of the soil bin’s depth restrictions. Models’ development was also limited to the following constant inputs; placement depth ($D$ = 0.2 m) and discharge ($q$ = 2.39 l h^−1^ m^−1^). The wetted depth $Z$ is not entirely an independent variable as factors such as crop-specific root water uptake influence the vertical soil–water movement.

## Conclusions and recommendations

The study adopted the Buckingham $\pi$ theorem or dimensional analysis to develop, calibrate and validate models’ that simulate soil wetting geometries for MTI as a function of soil hydraulic conductivity ($k$), placement depth ($D$), emitter discharge ($q$), and soil water content ($V$). Soil texture significantly affected the wetting geometry under MTI. The silty clay loam models satisfactorily simulated the wetted width or lateral soil moisture movement. It, however, failed to simulate the wetted depth at an operating pressure range of 20–100 kPa, which translated to an operating discharge of 0.27–1.18 l h^−1^ m^−1^. It can be concluded that the models are prescriptive, i.e., they should be used for the soils and conditions that they were developed under.

The study also noted, judging from the wetting pattern of the fine-textured soil, there is potential in MTI to provide plants with water with minimized deep percolation losses. The study was done in a soil bin on bare homogenous soil. The researchers recommend the study be carried under field conditions for both cropped and un-cropped soils and test the suitability of the developed models. Furthermore, the experiment was carried on dry soil. Thus an investigation on the soil wetted pattern under an initially moist soil should be carried out and compared to the current study (Supplementary Information 1).

## Supplementary Information

Supplementary Information.

## Data Availability

All relevant data are within the paper.
