# Supplementary material for: Development and validation of a model for soil wetting geometry under Moistube Irrigation
Source: Sci Rep. 2022 Feb 17;12:2737. doi: 10.1038/s41598-022-06763-x (PMC8854397; doi:10.1038/s41598-022-06763-x)
Supplement: Supplementary file 1 — Supplementary Information. [file 41598_2022_6763_MOESM1_ESM.docx]

**Development and Validation of a Model for Soil Wetting Geometry under Moistube Irrigation**

T.L. Dirwai^1,2,4*^, A. Senzanje^2^, and t. Mabhaudhi^3^

^1^ Crop-, Soil-, and Climate Sciences, University of the Free State, P.O.BOX 339, Bloemfontein 9300, South Africa

^2^School of Engineering, University of KwaZulu-Natal, P. Bag X01, Pietermaritzburg, 3209, South Africa

^3^ Centre for Transformative Agricultural and Food Systems, School of Agricultural, Earth and Environmental Sciences, University of KwaZulu-Natal, Pietermaritzburg, P. Bag X01, 3209, South Africa

^4^ Varmac Consulting Engineers, Scottsville, Pietermaritzburg, 3209, South Africa

*Corresponding author: TLD ([dirwaitl@ufs.ac.za](mailto:dirwaitl@ufs.ac.za))

Correspondence to: TLD (tldirwai@gmail.com)

**Appendix I**


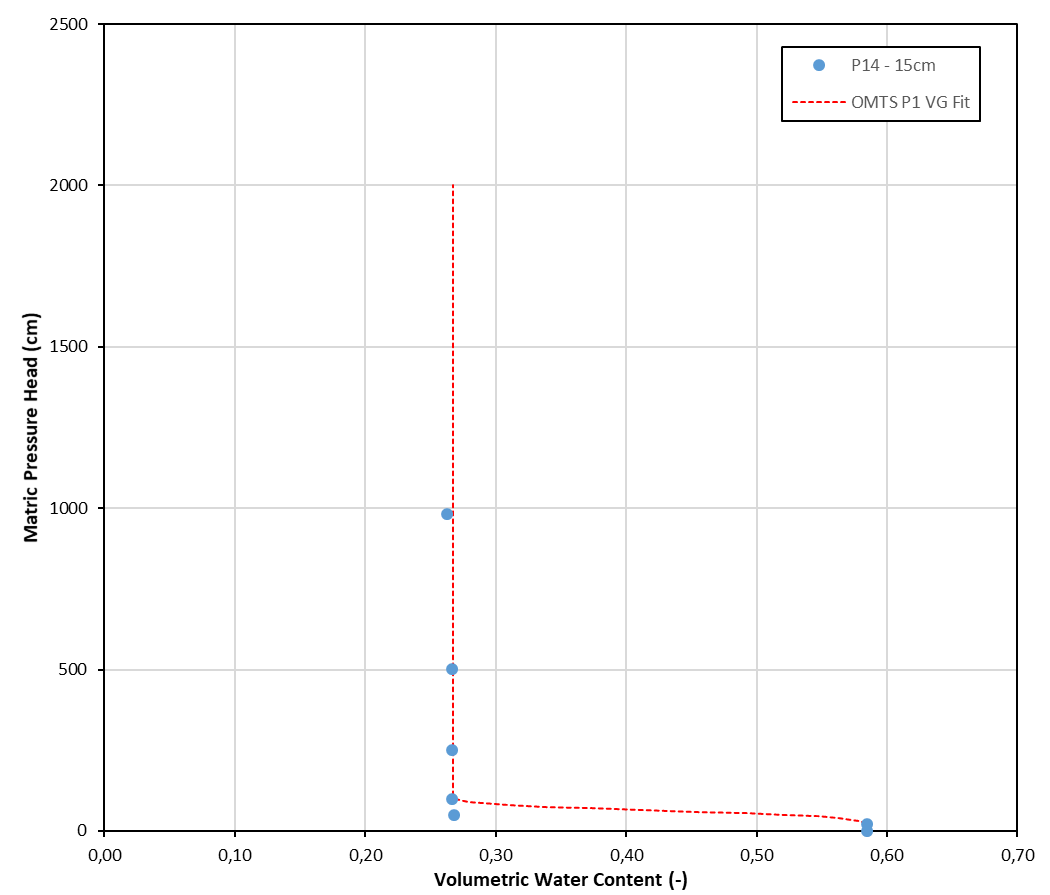


Figure 9 Soil matric – volumetric soil water content conversion chart for the silty clay loam soil
